# One‐year results of the Variation of Orthokeratology Lens Treatment Zone (VOLTZ) Study: a prospective randomised clinical trial

**DOI:** 10.1111/opo.12834

**Published:** 2021-05-15

**Authors:** Biyue Guo, Sin Wan Cheung, Randy Kojima, Pauline Cho

**Affiliations:** ^1^ Centre for Myopia Research School of Optometry The Hong Kong Polytechnic University Kowloon, Hong Kong Special Administrative Region China; ^2^ College of Optometry Pacific University Oregon USA

**Keywords:** BOZD, myopia control, orthokeratology, treatment zone

## Abstract

**Purpose:**

To present the 1‐year results of the Variation of Orthokeratology Lens Treatment Zone (VOLTZ) Study, which aims to investigate the myopia control effect of orthokeratology (ortho‐k) lenses with different back optic zone diameters (BOZD).

**Method:**

Children, aged 6 to <11 years, having myopia −4.00 D to −0.75 D, were randomly assigned to wear ortho‐k lenses with 6 mm (6‐MM group) or 5 mm (5‐MM group) BOZD. Data collection included changes in refraction, vision, lens performance and binding, ocular health conditions, axial length and characteristics of the treatment zone (TZ) area.

**Results:**

The 1‐year results of 34 and 36 subjects (right eye only) in the 6‐MM and 5‐MM groups, respectively, are presented. No significant differences in baseline demographics were found between the groups (*p* > 0.05). The first‐fit success rates, based on satisfactory centration at the 1‐month visit, were 100% and 94% respectively. Horizontal TZ size was 0.92 mm and 0.72 mm smaller in the 5‐MM group at the 6‐month and 12‐month visits, respectively (*p* < 0.05). At the 12‐month visit, no significant between‐group differences were found in the incidence of corneal staining (low grade only), lens binding and visual performance (all *p* > 0.05). Axial elongation was slower in the 5‐MM group (0.04 ± 0.15 mm) than the 6‐MM group (0.17 ± 0.13 mm) (*p* = 0.001). A significant positive correlation was observed between the horizontal TZ size and axial elongation (*r* = 0.36, *p* = 0.006).

**Conclusion:**

Clinical performance of the two ortho‐k lenses was similar, indicating that a smaller BOZD (5 mm) did not affect lens performance or ocular integrity. However, a smaller BOZD led to a reduced TZ, with retardation of axial elongation by 0.13 mm compared to conventional 6 mm BOZD ortho‐k lenses after one year of lens wear.

## Introduction

The prevalence of myopia in adults is higher in East Asian countries compared to other regions.^1^ This ethnic difference in myopia incidence is even more evident in the younger generation.^2^ Previous studies have reported a high proportion of myopia in 5–8 years old children in Japan (7 years old: 71.9%),^3^ Taiwan (7 years old: 25.4%)^4^ and Hong Kong (6–8 years old: 25.0%),^5^ compared with those of similar ages elsewhere, including Australia (6.7 years old: 1.4%)^6^, the USA (5–7 years old: 14.7%)^7^ and Poland (5–7 years old: 18%).^8^ The increasing prevalence of myopia worldwide^9^, ^10^ and the potential risk of developing sight‐threatening conditions^11^, ^12^ in highly myopic eyes has attracted attention to the importance of retarding myopia progression in children, thereby reducing the risks of later development of myopia‐related pathologies.

Recognition of the increasing prevalence of myopia and its associated complications has alerted the public to seek better solutions to control myopia.^13^ Currently, the most frequently used methods are optical devices and pharmaceutical interventions. Overnight orthokeratology (ortho‐k), which involves correcting myopia by reshaping corneal curvature overnight, has been shown to be effective in retarding eye elongation by 43%–63% in school‐aged children.^14^, ^15^, ^16^, ^17^, ^18^ Although the mechanism of ortho‐k in myopia control is not well understood, changes in peripheral defocus^19^ and aberration^20^ (especially spherical aberration and horizontal coma) have been hypothesised as explanations for its action. Both animal^21^ and human studies^22^ have demonstrated mid‐peripheral thickening of the epithelium after ortho‐k treatment, which was found to be positively correlated with increased spherical aberrations in a previous study^23^. Hiraoka *et al*.^24^ monitored 55 children aged 7.2–12.0 years and assessed ocular aberrations before and after 1 year of ortho‐k treatment. They reported that increased higher order aberrations were significantly correlated with axial elongation, with coma‐like aberration being the major contributing component. Faria‐Ribeiro *et al*.^25^ reported significantly higher 4th order spherical aberration and peripheral refraction (more myopic defocus) in subjects with larger pupils, which may be due to a greater area being exposed to higher order aberrations and peripheral refraction.

Lau *et al*.^26^ analysed retrospective data of 103 children treated with ortho‐k and found a negative correlation between the change in spherical aberration and axial elongation. They suggested that increases in higher order aberrations may play a role in the retardation of axial elongation in ortho‐k.

During ortho‐k treatment, the degree of myopia is reduced by flattening the central cornea. This central flattened zone is referred to as the treatment zone (TZ).^27^, ^28^ It has been hypothesised that a reduced treatment zone following ortho‐k creates increased peripheral refractive power and higher spherical aberration, which may further retard myopia progression.^28^ Some studies have reported the use of novel ortho‐k designs to enhance exposure to peripheral defocus and higher order aberrations by altering the relationship between the pupil size and the effective ortho‐k TZ.^27^, ^28^, ^29^ These lenses are designed with a reduced back optic zone diameter (BOZD), to achieve a smaller treatment area. Another study, conducted by Marcotte‐Collard *et al*.,^30^ compared the TZ characteristics of ortho‐k lenses with different designs, but having the same BOZD of 6 mm. Analysis of retrospective data from 64 subjects in each group using the 4‐zone Paragon CRT (Paragon Vision Sciences, paragonvision.com) and 5‐zone Dreamlens (DreamLens, dreamlens.com) revealed that the horizontal TZ size was larger in the 4‐zone group with a mean difference of 0.70 mm (*p* < 0.001). In another study investigating the modifying effect of reduced BOZD on the treatment area, lenses of each design were worn for two weeks. Subjects were first fitted with conventional 6 mm BOZD BE lenses (Capricornia Contact Lens, capcl.com.au) and then refitted with 5 mm BOZD BE lenses after a 2‐week wash‐out period.^27^ The authors observed that reducing BOZD did not change the peripheral refraction or corneal refractive power along the horizontal meridian in corneal topography. In a recent study by Carracedo *et al*.,^28^ 12 healthy adults (18 eyes) were randomly fitted with Paragon CRT lenses with either 5 or 6 mm BOZD for two weeks, and then, following a 2‐week washout period, switched to the other BOZD lenses for a further two weeks. A statistically smaller TZ (2.8 ± 0.2 mm), measured by Oculus Pentacam (Oculus, oculus.de), was observed in the 5 mm BOZD lenses compared with the 6 mm BOZD (3.1 ± 0.1 mm) lenses (*p* = 0.02). Greater central corneal flattening and greater mid‐peripheral steepening, as well as greater higher‐order aberrations, were observed with the smaller TZ lenses. Gifford *et al*.^29^ randomly assigned 16 adults to wear standard (PJ design, Capricornia Contact Lens, capcl.com.au) or test design (reduced BOZD by 0.5 mm) ortho‐k lenses for seven days with each design, with a one week wash‐out period in between. Calculation of the refractive power from raw data using a Medmont topographer (medmont.co.au), revealed the test group had a smaller horizontal TZ (4.78 ± 0.37 mm) compared to the standard group (5.70 ± 0.37 mm), but no changes were found in peripheral refraction. However, to date, there do not appear to be any published studies investigating how the TZ affects myopia control in ortho‐k treatment. Of the three studies investigating smaller BOZD ortho‐k lenses, Kang *et al*.^27^ and Gifford *et al*.^29^ concentrated on optical and refractive changes without discussing the performance or safety of smaller BOZD lenses. No significant difference in comfort and vision was reported by Carracedo *et al*.^28^ between the two designs, but less corneal staining was observed with the 6 mm BOZD lenses (37.5% vs 62.5%). However, it should be noted that the subjects in these studies were monitored for no more than two weeks. The current paper reports first‐fit success rate, lens performance, safety and most importantly, the effect of 5 mm BOZD lenses on axial elongation over a 12‐month period.

The Variation of Orthokeratology Lens Treatment Zone (VOLTZ) Study is a 2‐year myopia control study conducted at The Hong Kong Polytechnic University. This paper describes the study design and presents the 1‐year results. Essential clinical data were analysed, including axial length changes, ocular health status and lens performance. Other data collected for the longitudinal study will be analysed and reported at a later stage.

## Methods

### Study design

This 2‐year, randomised, double‐masked study was approved by the Ethics Committee of The Hong Kong Polytechnic University, registered with ClinicalTrials.gov (NCT03191942) and conducted at The Optometry Clinic of The Hong Kong Polytechnic University following the tenets of the Declaration of Helsinki. The study aims to compare myopia retardation, in terms of axial elongation, in two groups of young children wearing ortho‐k lenses of the same design, but different BOZD (conventional BOZD of 6 mm vs reduced BOZD of 5 mm). The primary outcome is axial elongation over two years.

### Subjects

Subjects were recruited according the criteria listed in *Table* *1* through advertisements in local newspapers and recommendations from existing subjects. All subjects gave assent and their parents or guardians signed a written consent before participation. Subjects were randomised to be fitted with 6 mm (control) or 5 mm (test) BOZD ortho‐k lenses in both eyes. They were provided with complimentary care solutions (*Table* *2*) and required to follow the instructions and replace the solutions and lens cases monthly. They were requested to wear lenses for eight hours overnight, except under the following conditions: sickness and/or taking medication, abnormal ocular signs/symptoms, including, but not limited, to pain, redness, itchiness or discomfort after lens insertion, which cannot be relieved after three attempts at reinsertion after rinsing. They were warned of the potential risks of infection/inflammation, with microbial keratitis being the worst case scenario, and were reminded to attend regular aftercare visits and follow instructions strictly to reduce the risks of complications before they consented/assented to participate in the study.

**Table 1:** Subject criteria for the Variation of Orthokeratology Lens Treatment Zone (VOLTZ) Study

Inclusion criteria	Exclusion criteria
Age: 6 to <11 years Chinese ethnicity (both parents) Myopia between −4.00 D to −0.75 D Astigmatism axes 180 ± 30: ≥−2.50 D other axes: ≥−0.50 D Less than 1.00 D difference in spherical equivalent between the two eyes Best corrected logMAR visual acuity 0.10 or better in both eyes Symmetrical corneal topography with corneal toricity less than 2.00 D in either eye Normal ocular health other than myopia Agree to be randomized and to attend the scheduled and aftercare visits Fulfil requirements for lens handling procedures	History of myopia control treatment (e.g., soft or rigid contact lenses, bifocal or multifocal spectacles, atropine eye drops) Strabismus or amblyopia Systemic condition which might affect refractive development (e.g., Down syndrome, Marfan syndrome), or ocular conditions which might affect refractive error (e.g., cataract, ptosis) Contraindications to contact lens wear and ortho‐k: history of ocular inflammation or infection, corneal dystrophy Poor compliance with scheduled visits Poor compliance with lens wear, including poor lens handling, poor vision and/or ocular response after lens modification

**Table 2:** Orthokeratology lenses and care solutions

KATT Lenses	Precision Technology Services (ptsoptics.com)
Material	HDS100
Oxygen permeability (barrer)	100
Design	Four‐zone Spherical/Toric reverse geometry
Back optic zone diameter (mm)	6.0 (conventional) or 5.0 (reduced BOZD)
Lens diameter (mm)	10.2/10.6/11.0/11.2
Central thickness (mm)	0.20
Back vertex power (D)	+0.50
**Contact lens solutions**	**Ophtecs Corporation (ophtecs.com)**
Cleaning	O2 Daily Care Solution
Rinsing	Cleadew Dissolving & Rinsing Solution
Disinfecting	Cleadew GP Advanced Care System^†^
Unidose artificial tears	Tiare W artificial tears

^†^ This is a povidone‐iodine based solution, neutralized by a tablet containing sodium sulphite and proteolytic enzyme.

### Randomisation and masking

Randomisation to use ortho‐k lenses with 6 mm BOZD (KATT BE Free, Precision Technology Services, ptsoptics.com) (6‐MM group) or 5 mm BOZD (KATT MC, Precision Technology Services, ptsoptics.com) (5‐MM group) in a 1:1 ratio was performed using a spreadsheet generator (Excel, Microsoft, microsoft.com), after subjects and their parents/guardians had met the criteria for lens handling and care procedures. The sequencing and allocation of randomisation was performed by the same practitioner involved in subject care and clinical follow‐up. Subjects were masked from the lenses used during the study period. Primary outcomes, which included axial length measurement and objective refraction after cycloplegia, were performed by a masked examiner.

### Interventions

The subjects were fitted with KATT lenses (*Table* *2*). The lens parameters were determined using computer‐aided software. A lens was considered as an unsuccessful fit when poor corneal response and/or clinically significant lens decentration was observed: significant decentration (>1.0 mm) or moderate decentration (>0.5 mm) with associated symptoms, such as compromised vision and/or affected ocular integrity.^31^ Lenses were replaced on an annual basis and/or when any of the following modifications was indicated: (a) residual myopia exceeded 0.50 D on two consecutive visits, (b) unaided visual acuity was worse than 0.18 LogMAR in either eye, (c) clinically significant lens decentration.

### Examination schedules and procedures

Potential subjects were examined at the screening visit. Eligible subjects were taught lens handling and care procedures. Baseline data collection visits were arranged after arrival of the new lenses. Lenses and care products were dispensed at the baseline visit and all subjects were required to attend regular aftercare visits after the first‐overnight lens wear (early morning visit), followed by weekly visits during the first month and every three months thereafter over the 2‐year period, to evaluate the lens performance and ocular conditions during ortho‐k wear. Unscheduled visits and/or referrals to ophthalmologists were arranged if necessary.

Cycloplegic examinations, at baseline, at 1‐month and at each 6‐monthly data collection visit, were conducted within ±2 h compared to the timing of the baseline visit to minimise diurnal variation. Cycloplegia was achieved by instilling two drops of 1% cyclopentolate, with a 5‐min interval between drops. Full cycloplegia, after at least 30 min, was defined as no pupil reaction to light and less than 2 D accommodation measured using an RAF ruler.

Before cycloplegia, high contrast (100%) unaided entrance visual acuity (UVA) and best corrected visual acuity (BCVA) were assessed using Early Treatment Diabetic Retinopathy Study charts (Precision Vision, precision‐vision.com) under normal lighting. Subjects or parents, who had received instructions prior to lens wear, were asked to grade the severity of lens binding from Grade 1 to Grade 4, using the grading scale defined elsewhere,^32^ each morning before lens removal. Corneal topography was measured using the Medmont E300 Topographer (Version 6.1.2, medmont.co.au). Tangential subtractive maps obtained by subtracting the post‐treatment map from the baseline map were used to evaluate lens centration. A decentration of more than 1.0 mm was considered as clinically significant (since previous studies reported mean TZ decentration of 0.6^33^ to 0.9^34^ mm on eyes with corneal toricity of less than 1.50 DC) and lens modification was deemed necessary. Lens adjustment was also necessary if moderate decentration (0.50 to 1.00 mm) was associated with compromised vision (UVA worse than 0.18 LogMAR) or corneal staining (Grade 2 or above).

After cycloplegia, subjective and objective refraction (Shin‐Nippon 5500K, Ajinomoto Trading, ajitrade.com/en) were assessed. Both the objective refraction and the axial length (IOLMaster 500, Carl Zeiss Meditec, zeiss.com) were performed by a masked examiner. Five axial length measurements with a signal‐to‐noise ratio of more than five and a maximum between‐difference of 0.02 mm were averaged for data analysis.

### Determination of treatment zone

**Figure 1:**
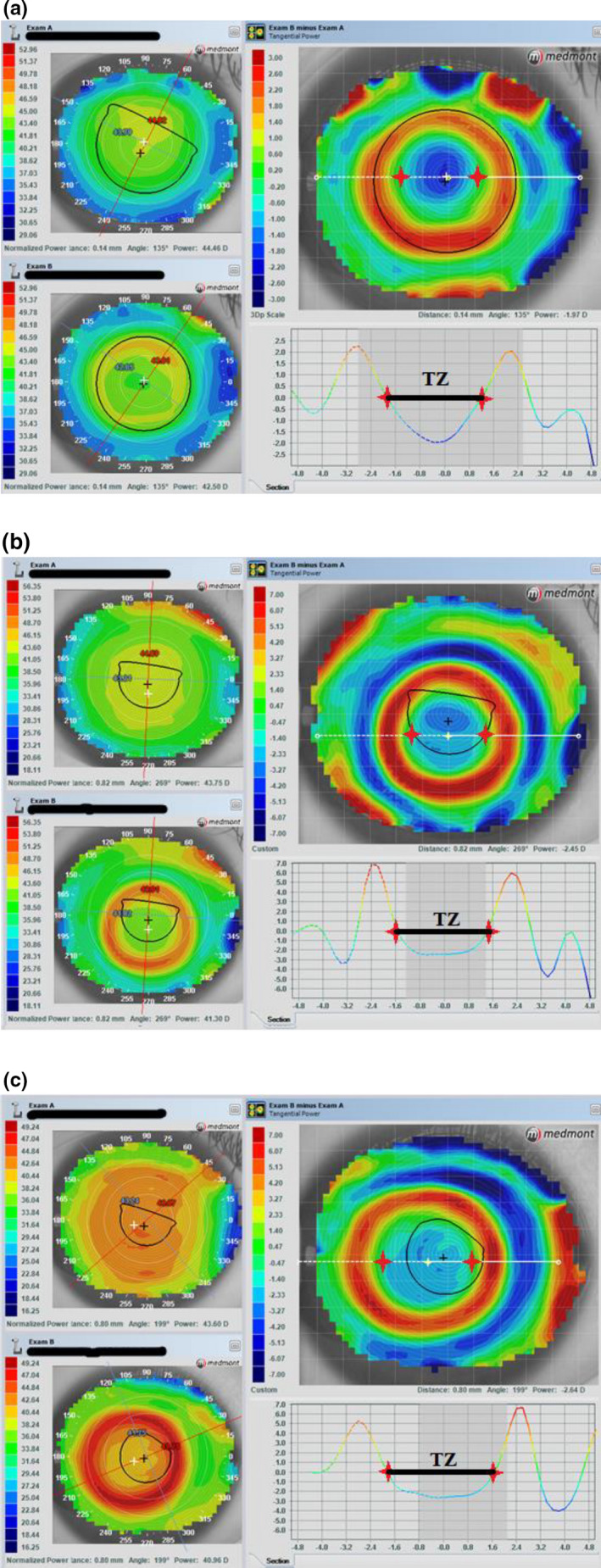
Illustration of treatment zone determination with/without decentration. (a) no/mild decentration (b) inferior decentration (c) lateral decentration.

Treatment zone was defined as the area enclosed by points of zero dioptric changes on the tangential subtractive topography maps. A cross‐sectional horizontal line was placed across the approximate elliptical centre of the TZ, and both TZ size and TZ central dioptric change (TZ‐CDC) were determined along this line. *Figure* *1* illustrates the determination of TZ when decentration was noted. The cursor was placed on the subtractive maps where the dioptric change was as close to zero as possible, with a tolerance of ±0.10 D at both end points of the line segment, allowing a maximum deviation of 0.20 D. The TZ‐CDC was defined as the dioptric change at the point assumed to be the geometric centre of the zone, which may not necessarily be the point with the greatest dioptric change. The value was shown when the cursor was hovered over the approximate geometric centre of the elliptical TZ. Measurements were performed by the same examiner following training and experience of its use. For each subject, only horizontal TZ size and TZ‐CDC of the right eye were determined at the 6‐ and 12‐month visits. Pre‐ and post‐ortho‐k topographical maps of 30 randomly selected subjects (15 in each group), coded by an independent person not involved in this study, were also used to investigate repeatability of measurements. The measurements were made on two separate days and the intra‐examiner intraclass correlation coefficient (ICC) and coefficient of repeatability (CR) were determined.

### Sample size

A calculated sample size of 24 in each group completing the study would provide 80% power to detect a change of 0.18 mm (0.50 D) in axial length between the two groups over two years, with a 5% level of significance, based on the SD of 0.25 mm from the ROMIO study.^14^ A minimum of 30 subjects were recruited in each group, assuming a 20% drop‐out rate over the two years study period.

### Statistical analysis

For the 1‐year data, statistical analyses were performed using SPSS version 25.0 (IBM, ibm.com). Normality of the measurements was determined by the Shapiro‐Wilk test. For normally distributed data, unpaired t‐tests were used to test the differences between the two groups. For data with non‐normal distribution, Mann‐Whitney tests were performed. Gender composition, grading of lens binding and lens fit success rate were compared between groups using the Chi‐square test. Overall correlation between TZ and axial elongation was evaluated using Pearson correlation coefficient. For each analysis, a 2‐tailed model was utilised and a significance level of 0.05 adopted. Only the data from the right eyes of each subject were analysed. The intra‐examiner ICC was calculated using the SPSS reliability analysis (two‐way random model with absolute agreement) and CR defined as $1.96\times \sqrt{\left(2S^{2}\right)}$ (*S* = within measurement SD).^35^

## Results

**Figure 2:**
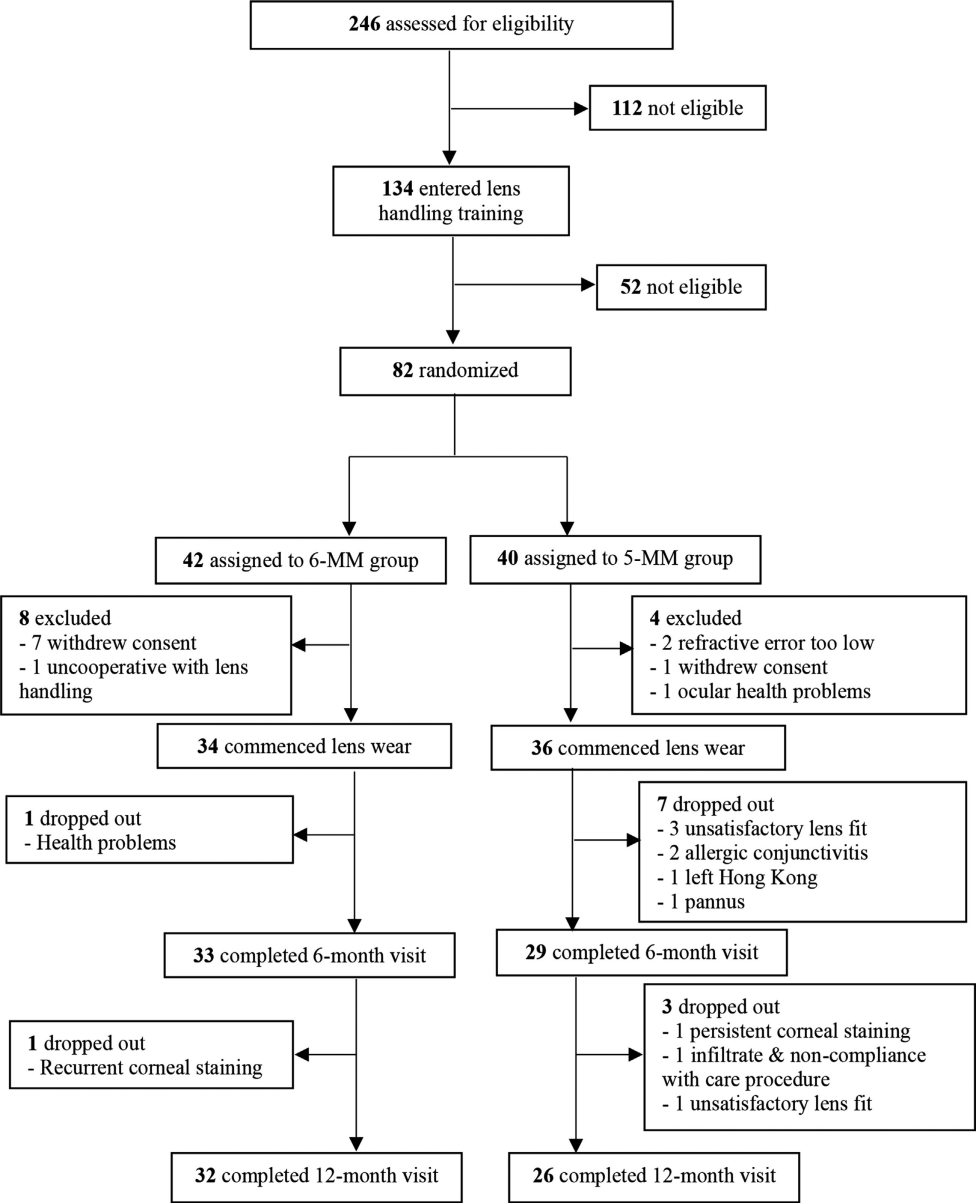
Flowchart showing subject recruitment and dropouts. (6‐MM, 5‐MM – wearing orthokeratology lenses of back optic zone diameter 6 and 5 mm, respectively.

A total of 82 subjects passed the recruitment criteria and were randomised to the 6‐MM and 5‐MM groups; 34 subjects and 36 subjects, respectively, satisfied the inclusion criteria after examination and commenced lens wear (*Figure* *2*). Two and 10 subjects from the 6‐MM and 5‐MM groups, respectively, dropped out of the study, and 32 and 26 subjects, respectively, completed the 12‐month visit. Demographics and baseline data, including age, gender, cycloplegic subjective refraction [myopia, astigmatism, spherical equivalent refraction (SER)], BCVA, and axial length, did not differ significantly between the two groups (*Table* *3*), or between drop‐outs and completed subjects in both groups (*p* > 0.05).

**Table 3:** Demographics and baseline data of orthokeratology subjects (median [range] or mean ± standard deviation) presented for non‐normally distributed and normally distributed data, respectively)

	6‐MM group		5‐MM group	
	All (*n* = 34)	Completed (*n* = 32)	All (*n* = 36)	Completed (*n* = 26)
Age (years)	9.16 ± 0.98	9.16 ± 1.00	9.32 ± 1.11	9.16 ± 1.22
Male/Female	13/21	12/20	15/21	9/17
Axial length (mm)	24.45 ± 0.71	24.44 ± 0.73	24.54 ± 0.77	24.60 ± 0.80
Spherical refraction (D)	−2.43 ± 0.92	−2.42 ± 0.94	−2.44 ± 0.80	−2.43 ± 0.83
Refractive astigmatism (D)	−0.25 [−1.25 to 0.00]	−0.25 [−1.25 to 0.00]	−0.50 [−1.50 to 0.00]	−0.50 [−1.50 to 0.00]
SER (D)	−2.62 ± 1.02	−2.62 ± 1.04	−2.68 ± 0.81	−2.68 ± 0.85
BCVA (logMAR)	−0.03 [−0.10 to 0.06]	−0.04 [−0.10 to 0.06]	−0.02 ± 0.05	−0.02 ± 0.06

6‐MM, using orthokeratology lenses of BOZD 6 mm; 5‐MM, using orthokeratology lenses of BOZD 5 mm; SER, spherical equivalent refractive error; BCVA, best corrected visual acuity; n, number of subjects.

### Residual refraction, visual acuity and first‐fit success rate

On average, SER (pre‐cycloplegic) reduced by 68% and 75% after the first‐night wear in the 6‐MM and 5‐MM groups, respectively (between groups, *p* = 0.24), reaching 105% and 110% at the end of the first month (between groups, *p* = 0.30). The first‐fit success rate, based on lens centration, at the 1‐month visit was 100% (34/34) in the 6‐MM group and 94% (33/35) in the 5‐MM group.

Within the first year, four subjects from the 5‐MM group failed to achieve acceptable lens fit and dropped out from the study. Residual cycloplegic subjective SER was significantly more hyperopic in the 5‐MM group compared to the 6‐MM group at the 6‐ and 12‐month visits, with differences of 0.44 D and 0.47 D, respectively (*p* ≤ 0.005), which reached clinical significance (*Table* *4*). Mean change in SER did not differ significantly between the two groups at either of the two visits (*p* ≥ 0.05) and no significant between‐group differences were found in BCVA and UVA (*p* ≥ 0.16).

**Table 4:** Residual cycloplegic subjective refraction and visual acuity measurements at 6‐month and 12‐month visits (median [range] or mean ± standard deviation) presented for non‐normally distributed and normally distributed data, respectively)

	Refractive astigmatism (D)	Change in astigmatism (D)	SER (D)	Change in SER (D)	BCVA (logMAR)	UVA (logMAR)
6‐month						
6‐MM group (*n* = 33)	−0.25 [−1.25 to 0.00]	−0.03 ± 0.41	0.09 ± 0.46	2.73 ± 1.02	−0.04 [−0.10 to 0.18]	0.02 ± 0.09
5‐MM group (*n* = 29)	−0.25 [−1.25 to 0.00]	0.25 [−0.50 to 1.50]	0.53 ± 0.65	3.27 ± 1.09	0.00 [−0.10 to 0.26]	0.02 [−0.10 to 0.34]
*p*	0.20	0.07	0.003^*^	0.05	0.16	0.40
12‐month						
6‐MM group (*n* = 32)	−0.50 [−1.00 to 0.00]	−0.06 ± 0.42	0.10 ± 0.51	2.72 ± 1.01	−0.02 ± 0.06	0.02 [−0.10 to 0.36]
5‐MM group (*n* = 26)	−0.25 [−1.25 to 0.00]	0.03 ± 0.46	0.57 ± 0.66	3.25 ± 1.17	−0.01 ± 0.06	0.03 ± 0.08
*p*	0.79	0.43	0.004^*^	0.07	0.75	0.98

6‐MM, using orthokeratology lenses of BOZD 6 mm; 5‐MM, using orthokeratology lenses of BOZD 5 mm; SER, spherical equivalent refraction, BCVA, best corrected visual acuity, UVA, uncorrected visual acuity, n, number of subjects, *p*, probability value of unpaired‐*t* or Mann‐Whitney *U* tests for difference between groups.

* Statisticaly significant differences between groups.

### Treatment zone size, central dioptric change, and axial length changes

Intra‐examiner (masked) ICC values indicated high repeatability of TZ size and TZ‐CDC measurements (0.992 and 0.998, respectively) and CR obtained were 0.19 mm and 0.17 D, respectively. Horizontal TZ size was significantly smaller in the 5‐MM group with a difference of 0.92 mm [95% confidence interval (CI): 0.74 to 1.10 mm] (*p* < 0.001) and 0.72 mm (*p* = 0.04) at the 6‐ and 12‐month visits, respectively (*Table* *5*). Horizontal TZ‐CDC was significantly more negative in the 5‐MM group at both the 6‐month (mean difference: 0.47 D, 95% CI: 0.06 to 0.87 D, *p* = 0.02) and 12‐month visits. TZ size and TZ‐CDC were not significantly different between the two visits for either group of subjects. With pooled data (combining the two groups), horizontal TZ size was positively correlated with horizontal TZ‐CDC at both 6‐month (*r *= 0.46, *p* < 0.001) and 12‐month (*r* = 0.31, *p* = 0.02) visits, that is, TZ‐CDC became more negative with smaller TZ size.

**Table 5:** Treatment zone (TZ) measurements and association with axial elongation at different visits (median [range] or mean ± standard deviation) presented for non‐normally distributed and normally distributed data, respectively

	6 months		12 months	
	TZ size (mm)	TZ‐CDC (D)	TZ size (mm)	TZ‐CDC (D)
6‐MM group	*n* = 33	*n* = 33	*n* = 32	*n* = 32
	3.59 ± 0.39	−2.45 ± 0.84	3.47 ± 0.52	−2.47 [−4.92 to −1.59]
5‐MM group	*n* = 29	*n* = 28^†^	*n* = 26	*n* = 25^†^
	2.67 ± 0.29	−2.92 ± 0.71	2.75 [1.75 to 3.15]	−3.00 ± 0.84
*p*	<0.001^*^	0.02^*^	<0.001^*^	0.04^*^
Association with axial elongation	*r* = 0.39	*r* = 0.22	*r* = 0.36	*r* = 0.17
	*p*’ = 0.002^*^	*p*’ = 0.09	*p*’ = 0.006^*^	*p*’ = 0.20

6‐MM, using orthokeratology lenses of BOZD 6 mm; 5‐MM, using orthokeratology lenses of BOZD 5 mm; *n*, number of subjects; *p*, probability value of unpaired‐t or Mann‐Whitney U tests for the difference between groups; *p*’, probability value for the Pearson’s correlation, r, Pearson’s correlation coefficient.

^†^ Treatment Zone‐Central Dioptric Change (TZ‐CDC) of two subjects (one at 6‐month and one at 12‐month) were excluded due to presence of central island.

* Statisticaly significant differences between groups.

**Figure 3:**
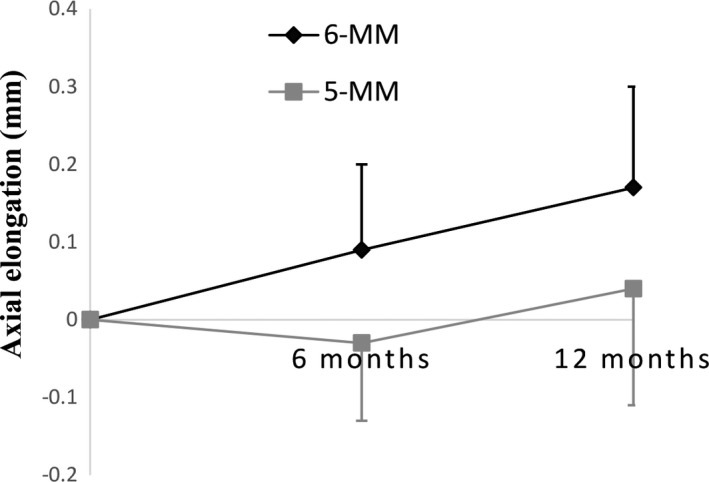
Axial elongation from baseline over 12 months in the two groups of orthokeratology subjects. Error bars represents one standard deviation. (6‐MM, 5‐MM – wearing orthokeratology lenses of back optic zone diameter 6 and 5 mm, respectively).

Overall, compared to the 6‐MM group, axial elongation was significantly less in the 5‐MM group, with a difference of 0.12 mm (95% CI: 0.06 to 0.17 mm) and 0.13 mm (95% CI: 0.06 to 0.21 mm) at the 6‐month and 12‐month visits, respectively (*p* ≤ 0.001). However, the difference in axial elongation between the two groups was only significant during the first six months; axial elongation in the subsequent six months, that is, between the 6‐month and 12‐month visits, was not significantly different between the two groups (*Table* *6* and *Figure* *3*). A significant correlation was found between horizontal TZ size and axial elongation at the 6‐month (*r* = 0.39, *p* = 0.002) and 12‐month (*r* = 0.36, *p* = 0.006) visits, but no significant correlation was found for TZ‐CDC (*p* > 0.05) (*Table* *5*).

**Table 6:** Axial length changes at 6‐month and 12‐month visits (median [range] or mean ± standard deviation) presented for non‐normally distributed and normally distributed data, respectively

	AL 6 M‐BL (mm)	AL 12 M‐BL (mm)	AL 12 M‐6 M (mm)
6‐MM group	0.09 ± 0.11	0.17 ± 0.13	0.09 ± 0.06
	*n* = 33	*n* = 32	*n* = 32
5‐MM group	−0.03 ± 0.10	0.04 ± 0.15	0.05 [−0.05 to 0.21]
	*n* = 29	*n* = 26	*n* = 26
*p*	<0.001^*^	0.001^*^	0.19

6‐MM, using orthokeratology lenses of BOZD 6 mm; 5‐MM, using orthokeratology lenses of BOZD 5 mm; AL, axial elongation; BL, baseline; 6 M, 6‐month; 12 M, 12‐month; *p*, probability value of unpaired‐*t* or Mann‐Whitney *U* tests for difference between groups, *n*, number of subjects.

* Statistically significant differences between groups.

### Adverse events

Reports of halos, by one (3%) 6‐MM subject and three (8%) 5‐MM subjects at the first overnight visit (*p* > 0.05), subsided over the following two weeks, without causing any visual discomfort. One subject was found to have a 0.4 mm neovascularization with pannus in the inferior nasal cornea of the right eye at the 1‐month visit, so lens wear was discontinued. The subject was followed‐up by the same examiner for six months to monitor the progression of the neovascularization after ceasing lens wear and there was no change over time.

The incidence of lens binding at various visits is shown in *Table* *7*. No significant difference in incidence of lens binding was observed between the two groups at any visit (*p* = 0.24 to 0.73). The severity of lens binding reported also did not differ significantly between groups at any visit (*p* ≥ 0.06). Lens binding‐associated Grade 1 corneal staining was observed in only three (9%) 6‐MM subjects at the first month, at 6‐month and 12‐month visits, respectively. After reinforcement of correct handling techniques, no further staining was observed at subsequent visits. Non‐clinically significant corneal staining (≤Grade 2) was observed in some subjects during the first year of lens wear, but the incidence of staining was not statistically different between the groups at any of the visits (*p* ≥ 0.11) (*Table* *7*).

**Table 7:** Summary of adverse events within the first year of lens wear in subjects wearing orthokeratology lenses having back optic zone diameters of 6 and 5 mm

		BL	FO	1 M	6 M	12 M
6‐MM	Lens binding	/	53%	47%	36%	38%
	Corneal staining	15%	21%	18%	21%	34%
	Infiltrate	/	/	/	0	9%
	Microcysts	/	/	/	0	3%
		*n* = 34	*n* = 34	*n* = 34	*n* = 33	*n* = 32
5‐MM	Lens binding	/	39%	43%	28%	27%
	Corneal staining	17%	11%	14%	7%	23%
	Infiltrate	/	/	/	3%	12%
		*n* = 36	*n* = 36	*n* = 35	*n* = 29	*n* = 26

6‐MM, using orthokeratology lenses of BOZD 6 mm; 5‐MM, using orthokeratology lenses of BOZD 5 mm; BL, baseline; FO, first overnight; 1 M, 1‐month; 6 M, 6‐month​; 12 M, 12‐month.

Sterile corneal infiltrates were observed in seven subjects at the 6‐month and 12‐month visits, four in 5‐MM and three in 6‐MM groups, respectively (*Table* *7*). Of the subjects with infiltrates during the first year, all but one continued lens wear with stable ocular conditions after reinforcement of lens handling and change of lens care solutions (to multipurpose solution, daily cleaner and protein removal solution). One 5‐MM subject was non‐compliant, with severe protocol deviation in lens handling (untrained domestic helper assisted with lens removal), and was excluded from the study after the 9‐month visit. One (3%) 6‐MM subject showed Grade 2 microcysts^36^ at the 9‐month visit, which recurred at the 12‐month visit after resuming lens wear. Lens wear was terminated for this subject thereafter.

## Discussion

This paper presents first year results of a longitudinal study comparing treatment effects of 6 and 5 mm BOZD ortho‐k lenses and shows that reducing TZ is effective in increasing the effectiveness of myopia control in terms of axial elongation during the first six months.

The first‐fit success rates of the two lens designs were comparable: 100% and 94% 6‐MM and 5‐MM subjects achieved successful lens fit at the 1‐month visit, which was comparable to the 95% reported by Tan *et al*.^37^ (both eyes) using the same brand of 6 mm BOZD lenses. However, it should be noted that four subjects dropped out from the study (see *Figure* *2*) because the 5 mm BOZD lenses were unable to achieve a smooth and well‐centred TZ and/or good visual performance even after modifications to the lens parameters (e.g., loosen/tighten the alignment zone). All four subjects were able to achieve acceptable fitting with the 6 mm BOZD lenses and were referred for further ortho‐k management. The difference in lens fits may be related to the squeeze film forces underneath the back surface of the lens, although the exact mechanism is not fully understood.

There were no significant differences in clinical performance between the two groups, in terms of changes in SER, refractive astigmatism, BCVA, UVA or ocular health at either the 6‐month or 12‐month visits. Three subjects in the 5‐MM group reported halos after the first‐overnight visit, compared to only one in the 6‐MM group. Reports of halos were to be expected, given that the 5 mm BOZD is meant to reduce the TZ size. However, since the halos did not affect the subjects’ visual performance or daily activities, no intervention was undertaken, and the symptoms subsided after adaptation to lens wear.

Neither lens design resulted in any severe adverse events. The incidence of mild levels of corneal staining observed was comparable to previous studies.^32^, ^38^ Walline *et al*.^38^ reported mild corneal staining in about 60% and 30% of their subjects at the morning and afternoon visits, respectively. The pannus found in one 5‐MM subject at the 1‐month visit was unlikely to have been a result of lens wear. It was more likely to have been overlooked at the baseline visit as the clinical presentation of a remnant from an old event. It did not change in appearance even after lens wear was terminated for six months. Sterile infiltrates have been reported in previous studies with incidences of 1%^39^ and 0.8%,^40^ and temporary lens cessation was recommended. In the current study, four out of seven subjects with infiltrative response were willing to continue the project after full recovery; the remaining three stopped lens wear. The four subjects were switched to multipurpose solutions; two after the recurrence of an infiltrative response. The infiltrates observed here may be accompanied by punctate corneal staining and palpebral papillae in the upper lids, and were resolved by changing the lens care solutions which may indicate an allergic reaction rather than inflammation. The infiltrative responses may have resulted from inadequate rubbing and rinsing of solutions before lens insertion, solution allergies or non‐compliance with lens care regime (e.g., an untrained domestic helper handling lenses). Excluding one individual who was non‐compliant with the care regime, all subjects with an infiltrative response resumed lens wear after the condition subsided, and they were reminded of the proper lens care procedures.

Mild to moderate lens binding, without causing significant corneal problems, has been reported previously in ortho‐k lens wearers.^32^, ^41^ Chan *et al*.^41^ retrieved data from 108 clinic records and conducted a telephone interview with the parents of the children undergoing ortho‐k treatment, of whom 44% reported lens binding, with an incidence of 17% and 30% of subjects at the first‐overnight and 1‐month visits, respectively. All subjects in the current study were advised to apply artificial tears to loosen the lens prior to removal, if necessary, to reduce the risk of ocular insult caused by improper removal of a bound lens. The appropriate procedures to loosen a bound lens, either using artificial tears or through conjunctival manipulation, need to be reinforced. Severe lens binding that cannot be loosened using these methods may indicate a need to refit to a flatter alignment zone lens with increased eccentricity (e‐value).

Intra‐examiner TZ size and TZ‐CDC measurements indicated good repeatability, with high ICCs (>0.9). With respect to the CR (0.19 mm for TZ size), since the placido rings of the Medmont topographer are 0.24 mm apart, a 0.19 mm CR is satisfactory to detect real differences in the size of the TZ captured by the topographer. In addition, the difference between the two groups was substantial: 0.92 mm at 6‐month and 0.72 mm at 12‐month visits. For the TZ‐CDC, a CR of 0.17 D is not clinically significant.

The use of a 5 mm BOZD lens produced a significantly smaller horizontal TZ compared with the 6 mm BOZD lens at the 12‐month visits. The 0.72–0.92 mm difference in TZ size was comparable to the 0.92 mm difference reported by Gifford *et al*. using standard and reduced BOZD (by 0.5 mm) ortho‐k lenses (PJ design, Capricornia Contact Lens, capcl.com.au)^29^, and greater than that reported previously using Paragon CRT™ contact lenses (Paragon Vision Sciences, paragonvision.com) (6 mm: 3.1 ± 0.1 mm; 5 mm: 2.8 ± 0.2 mm, *p* = 0.02).^28^ The horizontal TZ‐CDC was significantly different between the groups at the 6‐month and 12‐month visits, being greater in the 5‐MM group, in which there was a higher power difference from the apex to the edge of the TZ. This may be associated with increased peripheral defocus, warranting further investigation. There is a significant positive association between TZ size and TZ‐CDC at both the 6‐month and 12‐month visits. This results in a greater TZ‐CDC with reduced TZ size, since the TZ‐CDC is presented in negative sign (dioptric changes).

At the 6‐ and 12‐month visits, the axial length changes were significantly smaller in the 5‐MM compared to the 6‐MM group and a significant positive correlation, although weak (*r* ^2^ = 0.15), was found between axial length changes and TZ size. The results suggested a stronger retardation in axial elongation using smaller BOZD (5‐MM) ortho‐k lenses, which led to a 0.33 mm smaller change in axial length after 1‐year of ortho‐k‐wear compared to historical spectacle‐wearing controls^14^ (0.04 mm vs 0.37 mm). This result was substantially improved in comparison with previous reports. Cho *et al*.^42^ monitored the myopia progression in 35 Asian children wearing ortho‐k lenses during the first year and found a 0.18 mm shorter axial elongation compared to retrospective controls (0.16 mm vs 0.34 mm). Walline *et al*.^18^ recruited 40 children, the majority of whom were White, for ortho‐k treatment and showed a slower increase in axial length of 0.15 mm at the 12‐month visit compared to historical control subjects (0.15 mm vs 0.30 mm). Santodomingo‐Rubido *et al*.^17^ fitted 31 White children, whose myopia progression was slower by 0.15 mm after one year of wear (0.22 mm vs 0.37 mm). Hiraoka *et al*.^43^ and Cho and Cheung^14^ recruited Japanese and Chinese children, respectively, and reported axial elongation of 0.19 mm vs 0.38 mm and 0.20 mm vs 0.36 mm in their ortho‐k subjects (myopia control effect of 0.19 mm and 0.16 mm). In the current study, the axial elongation was 0.17 mm for the 6‐MM subjects and 0.04 mm for the 5‐MM group, i.e., those wearing the reduced BOZD lenses exhibited a further retardation in axial elongation of 0.13 mm after one year of treatment, which reached clinical significance.

The aim of the VOLTZ study was to investigate whether axial elongation of myopic children could be slowed further if a novel lens design with a smaller BOZD was used. Hence, no single vision spectacle control subjects were recruited. Axial elongation after one year of conventional ortho‐k lens (6 mm BOZD) wear was comparable to that of the ortho‐k group from the ROMIO study^14^ (0.17 vs 0.20 mm, *p* = 0.38). Compared to the control group (spectacles) in the ROMIO study, myopia control, in terms of axial elongation was 89% (0.04 vs 0.37 mm), indicating a very substantial increase in myopia control using the 5 mm BOZD lens.

In this study, the improved effect mainly occurred during the first six months of treatment, as there was no significant difference in axial elongation between groups during the second six months of lens wear. This coincides with the lack of differences in changes of TZ size and TZ‐CDC during the second six months. Similar results were reported by Tan *et al*.,^44^ in which a combined ortho‐k and 0.01% atropine treatment was employed. The improved myopic control effect was only observed in the first six months, with a significant moderate correlation found between photopic pupil size and axial elongation in the combined therapy group only at the 6‐month time point, but not at 12‐months. The authors suggested a potential contribution of increased pupil size, which allows vision through a corneal surface with a larger change in higher order aberrations.^25^ Myopia control using ortho‐k together with atropine stabilised within 6‐month of lens wear and there was no subsequent change thereafter. In the current study, rather than increasing the pupil size, the TZ was made smaller using the 5 mm BOZD lenses. It is possible that this allows vision through a corneal surface with a larger change in higher order aberrations, hence achieving an improvement in myopia control. The results suggest a potential association between the myopia control effect and the relationship between pupil size and TZ size. Further studies and analysis are warranted to investigate this potential correlation.

In conclusion, ortho‐k lenses with a 5 mm BOZD achieved similar clinical performance to a 6 mm BOZD lens. All and 94% of subjects in the 6‐MM and 5‐MM groups, respectively, achieved satisfactory lens centration at the 1‐month visit. Four subjects from the 5‐MM group failed to achieve acceptable lens fit and dropped out from the study during the first year.

No clinically significant adverse effects were reported, except for more initial complaints of halos, which reduced with lens wear. Lens wear (either BOZD) was well accepted by the subjects. Reduced TZ ortho‐k lenses showed a further (substantial) retardation of axial elongation by 0.13 mm compared with conventional ortho‐k lenses. Since the majority of changes occurred within the first six months, a longer study period is warranted to investigate the treatment effect over time.

## Author contributions

**Biyue Guo:** Conceptualization (supporting); Data curation (lead); Formal analysis (lead); Investigation (equal); Methodology (equal); Project administration (equal); Resources (supporting); Software (equal); Writing‐original draft (lead); Writing‐review & editing (equal). **Sin Wan Cheung:** Conceptualization (supporting); Formal analysis (supporting); Investigation (supporting); Methodology (supporting); Project administration (supporting); Resources (supporting); Writing‐review & editing (supporting). **Randy Kojima:** Conceptualization (supporting); Writing‐review & editing (supporting). **Pauline Cho:** Conceptualization (lead); Formal analysis (supporting); Funding acquisition (lead); Investigation (equal); Methodology (equal); Project administration (equal); Resources (lead); Supervision (lead); Writing‐review & editing (equal).

## Disclosure

All authors, except R Kojima, have no proprietary interest in any of the products mentioned. R Kojima is a Clinical Research and Development Director for Precision Technology Services (Vancouver, Canada), a partner in the KATT Design Group (Vancouver, Canada) and a clinical advisor to Medmont International Pty, (Nunawading, Australia).
